# Tension, Free Space, and Cell Damage in a Microfluidic Wound Healing Assay

**DOI:** 10.1371/journal.pone.0024283

**Published:** 2011-09-06

**Authors:** Michael Murrell, Roger Kamm, Paul Matsudaira

**Affiliations:** 1 Department of Biological Engineering/Massachusetts Institute of Technology, Cambridge, Massachusetts, United States of America; 2 Departments of Biological and Mechanical Engineering/Massachusetts Institute of Technology, Cambridge, Massachusetts, United States of America; 3 Whitehead Institute for Biomedical Research, Departments of Biological Engineering and Biology/Massachusetts Institute of Technology, Cambridge, Massachusetts, United States of America; Dalhousie University, Canada

## Abstract

We use a novel, microfluidics-based technique to deconstruct the classical wound healing scratch assay, decoupling the contribution of free space and cell damage on the migratory dynamics of an epithelial sheet. This method utilizes multiple laminar flows to selectively cleave cells enzymatically, and allows us to present a ‘damage free’ denudation. We therefore isolate the influence of free space on the onset of sheet migration. First, we observe denudation directly to measure the retraction in the cell sheet that occurs after cell-cell contact is broken, providing direct and quantitative evidence of strong tension within the sheet. We further probe the mechanical integrity of the sheet without denudation, instead using laminar flows to selectively inactivate actomyosin contractility. In both cases, retraction is observed over many cell diameters. We then extend this method and complement the enzymatic denudation with analogies to wounding, including gradients in signals associated with cell damage, such as reactive oxygen species, suspected to play a role in the induction of movement after wounding. These chemical factors are evaluated in combination with the enzymatic cleavage of cells, and are assessed for their influence on the collective migration of a non-abrasively denuded epithelial sheet. We conclude that free space alone is sufficient to induce movement, but this movement is predominantly limited to the leading edge, leaving cells further from the edge less able to move towards the wound. Surprisingly, when coupled with a gradient in ROS to simulate the chemical effects of abrasion however, motility was not restored, but further inhibited.

## Introduction

The collective movement of epithelial sheets is critical to diverse physiological processes, from early development to homeostasis in the adult. In the drosophila embryo, cell sheets move collectively to coordinate dorsal closure [1]. In the mouse adult gastrointestinal mucosa, epithelial sheets move continuously along the crypt villus axis of the intestine [2], [3]. Thus, the collective motion of cell sheets is integral to the proper functioning of a wide variety of tissues. Epithelial sheets also move collectively in response to stress. In the wounded cornea for example, epithelial sheets have been shown to heal by sliding into the damaged area [4]. Therefore, wounding is a convenient and tractable assay for studying the collective motion of cell sheets in response to an artificial perturbation.

*In vitro* analyses of epithelial sheet migration have largely focused on wounding by scratching (e.g. with a pipette tip or a razor blade) to induce sheet motion that resembles movement observed in wounds *in vivo* [5], [6]. The wounding of an epithelial sheet however, exposes cells to many heterogeneous factors that potentially affect migration, and these effects are difficult to disentangle. Cells are not only exposed to free space, but the abrasion damages cells and releases chemical factors that induce changes in gene expression and cellular signaling [7], [8]. One hypothesis is that the directed movement of cell sheets that results from this wounding, either *in vitro* or *in vivo*, is caused by the sudden availability of permissive space available to leading edge cells, i.e. the cells closest to the free border [9]. The leading edge cells that spread out into the newly vacated space are then responsible for the motility of the rear, or submarginal cells by mechanically pulling them forward through E-cadherin based cell-cell contact [10]–[12]. However, it is unclear if the initial driving force for leading edge migration is due to the creation of free space, or the induction of signaling from cell damage due to scratching. Nor is it clear whether motility of the submarginal cells is caused by being pulled by the leading edge, or if significant tension exists across the epithelial sheet.

Previous efforts to separate the influence of cell damage from the creation of free space include the application of PDMS blocks or stencils as ‘ model’ wounds [13]–[15]. These methods utilize a barrier to confine cells physically, and upon its removal, cells are unconfined and free to migrate. While these methods significantly advance our understanding of the role of constraints in coordinated motion, the removal of constraints is not equivalent to a wound minus the effects of abrasion. First, barriers require a large, localized physical interaction with the epithelial sheet (i.e. removal of a macroscopic object at the border). This still induces cell death, albeit significantly less than would be induced by scratching [14]. Certainty of the isolation of free space therefore hinges critically on eliminating cell death but furthermore, in the absence of cell death, minimizing the physical trauma to cells at the border upon denudation. Many studies have shown that mechanical stress induces many changes in cell behavior [16], including cell migration [17]–[21]. Therefore prevention of cell death alone is insufficient to isolate the effects of free space. Second, the confinement of cells by the application of a barrier replaces what would otherwise have been cell-cell contacts with a cell-PDMS interface. Thus, the un-confinement, or removal of the barrier does not involve the breaking of cell-cell contact. With no cell-cell contacts at the (new) leading edge there may not have been the same balance of tension at this interface. The potential for leading edge cells to close the wound by pulling hinges critically on the existence of tension between cells in the sheet [22]. Therefore, we cannot assume that the broken cell-cell bonds that result from wounding, and the absence of cell-cell bonds during un-confinement are equivalent initial conditions to the onset of migration.

There have been notable efforts thus far in the development of a microfluidic assay to study cell migration. This includes the proof of concept that trypsin can be used to remove cells cultured in microchannels [23], and that migration can be assayed with this technology [24]. Furthermore, recent studies have used this method to investigate the role of VEGF signaling in endothelial wound closure [25]. However, it is not clear in the case of endothelial wound healing, whether wound closure is dominated by cell spreading at the border, or by net migration of the tissue. The wound was small and individual cell motion was not assayed. The extent that submarginal migration contributes to the movement of the cell sheet remains in question. Furthermore, quantitative roles for the many chemical responses that result from scratching on the migration of a cell sheet have not yet been identified.

An important element of the chemical environment created during wounding is the presence of reactive oxygen species (ROS), known to be associated with the promotion of migration during wound closure. Generated from intracellular 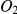, ROS are products of external stress, and are known to act as second messengers, coupling to intracellular signaling pathways [27]. Formed at the leading edge of a wounded sheet (within the first 3-4 rows of cells), ROS persists for minutes following wounding [13]. Added exogenously, their effects are highly concentration dependent. Low concentrations promote wound closure, while high concentrations are detrimental to migration [28], [29]. Hydrogen Peroxide (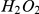) is a ROS which has been shown to differentially modulate growth and apoptosis, and regulates cell-cell adhesion and migration. The production of 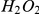 enhances migration through the degradation of the extracellular matrix by the activation of matrix metalloproteinases in fibroblasts [30]. Additionally, ‘ fibroblast-like’ phenotypes have been observed in mammary epithelial cells following prolonged exposure [31]. Brief exposure however, increases the expression of inter-cellular adhesion molecule ICAM-1 [32]. Thus, recapitulating the chemical environment of the wound with ROS would require precise spatial and temporal control over concentration.

We create a model wound in a microfluidic environment, where we can introduce gradients in proteins and small molecules sequentially. First, we flow trypsin to selectively remove cells enzymatically. The trypsin generates free space without inducing an abrasive cell death at the leading edge of the cell sheet. During trypsin flow, we quantify the rate of retraction of the sheet that occurs when cell-cell bonds at the leading edge are broken. To connect this macroscopic retraction to the molecular mechanisms that are known to generate tension between cells we create a gradient in actomyosin contractility by the localized delivery of blebbistatin instead of denudation by trypsin. As tension can manifest between individual cells [33], [34], we sought to quantify how tension manifests collectively, and how when broken, it affects the ability of the wound to heal. The migration of an enzymatically denuded sheet in a microfluidic chip is then assessed and contrasted with the migration of epithelial cells in the classical scratch assay. Finally, we couple denudation by trypsin with the subsequent delivery of gradients in 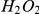 to recreate the spatial and temporal localization of ROS that exists in a wound healing assay as an analog to what is produced post abrasion.

We conclude that exposure to free space, in the absence of cell death, is sufficient to induce migration in the leading edge cells. The submarginal cells however, move slowly and are less persistent than in the classical scratch assay. Surprisingly, the addition of lysate or ROS to simulate the chemical effect does not restore rear motility, but hinders motility in both the leading edge and submarginal regions.

## Results

### Construction of a Model Wound

**Figure 1:**
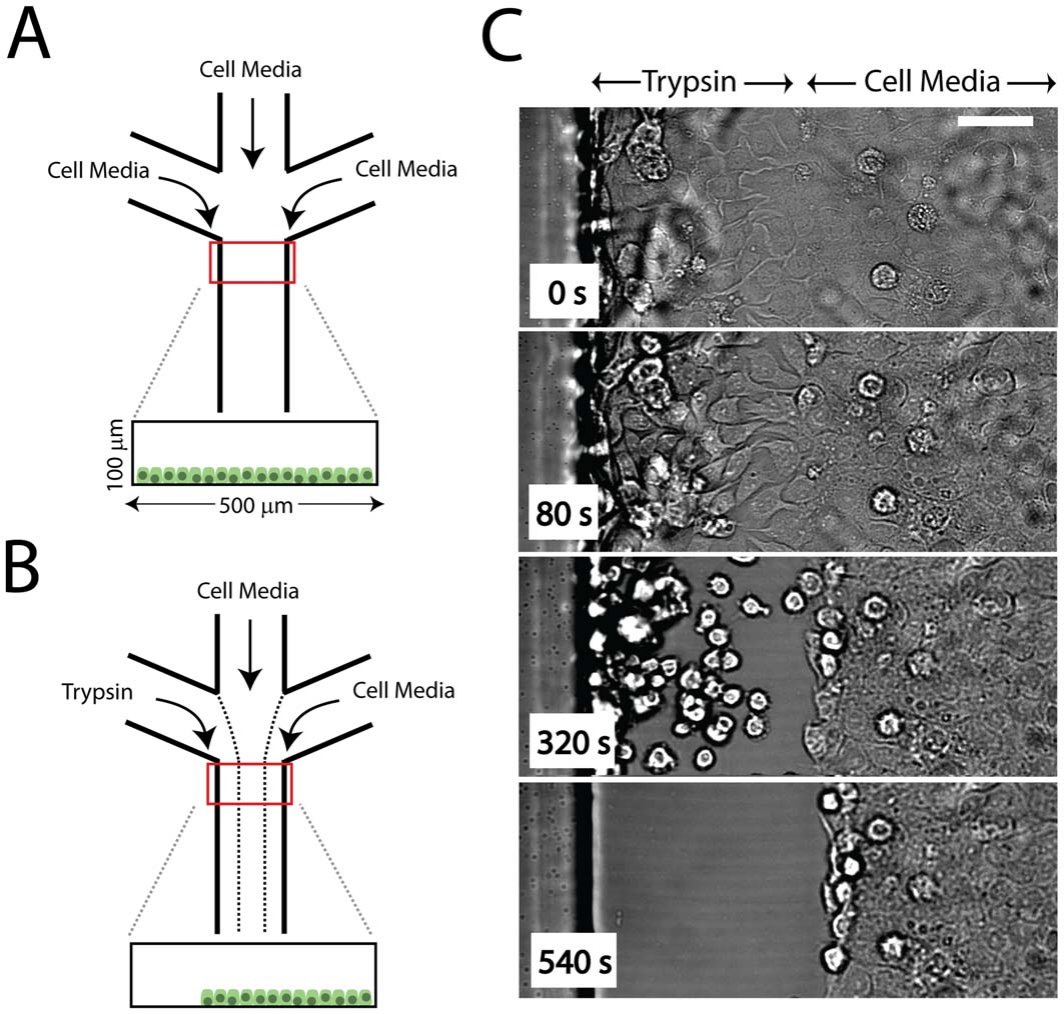
Experimental Design. (A) Schematic of Experimental Setup. Three inlets supply cell media to an epithelial sheet cultured in the 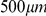 wide, 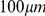 high channels. This is done under no flow. (B) After cells have reached confluency, 15 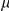
l/min flow (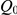) is applied, leading to three separated laminar streams. One stream contains 0.05% trypsin, while the other two contain cell media. This cleaves cells from the channel as shown in (C). On average, cleavage takes 5 min. Images are acquired in brightfield.

We utilize a simple microfluidic chip, comprised of three channels that merge into one (described in the Materials and Methods). Mouse mammary epithelial cells are cultured inside the channel until they reach confluency (Fig. 1A). To mimic the denudation by scratching, we denude space by the local delivery of trypsin. Trypsin is perfused through one of three inlets, with cell media purfused through the other two. Flow is laminar, and produces three separate streams which flow across the confluent epithelial sheet (Fig. 1B). Within minutes, cells exposed to trypsin are enzymatically cleaved from the surface (Movie S1), leaving behind the rest of the epithelial sheet which was exposed only to cell media (Fig. 1C).

**Figure 2:**
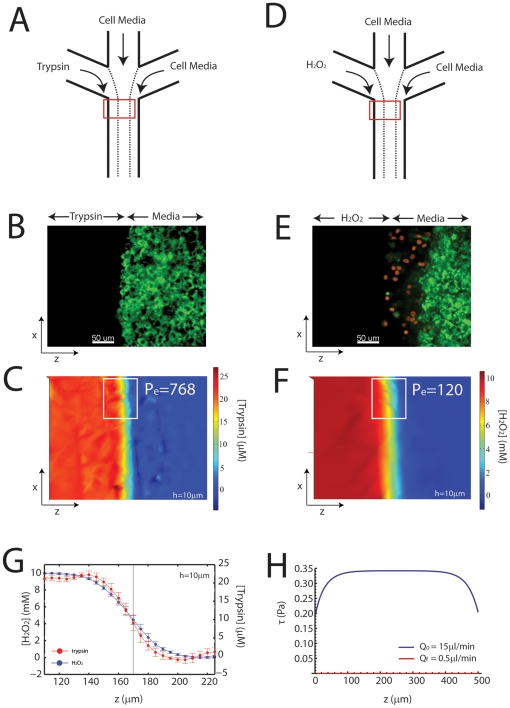
Composition of a Reconstituted Wound. (A) Schematic of Experimental Setup in (B) and (C). Cell media and trypsin merge to selectively cleave cells from a confluent epithelial sheet. (B) A gradient in trypsin (0.05% or 21.4
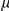
M) denudes the cell sheet, leaving cells viable as shown by a live stain (green). (C) Two-dimensional plot of trypsin concentration during denudation. The flow, 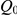, is 15
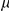
l/min for an average of 5 min. The diffusion coefficient for trypsin, 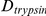 = 2.1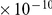 m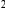/s was estimated with a molecular radius of 1.5 nm [50]. The white box refers to the region which is averaged to see a line plot perpendicular to the direction of flow in (G). (D) Schematic of the Experimental Setup in (E) and (F). 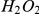 replaced trypsin after the cleavage of cells. (E) Delivery of 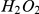 (10mM) after trypsin treatment creates an apoptotic border (1-4 rows of cells), and immerses the rest of the sheet in a non-apoptotic concentration. Green: alive, red: dead. (F) Concentration profile of 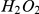 for 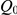 up to 15 min. The diffusion coefficient for 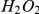, 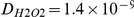 m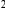/s was taken at 25C [51]. This diffusivity creates a larger mixing zone than the mixing zone for trypsin. The white box refers to the line plot in (G). (G) Line plot of trypsin and peroxide concentrations averaged over the x dimension in parts (C) and (F). The calculation is taken 10 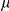
m from the coverslip, to approximate the concentration at the apical surface of the cells. (F) Shear calculation across the width of the channel (z) for the initial flow, 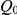, used for delivery of trypsin and 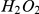, and final flow of pure media (no trypsin or peroxide) at 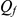 = 0.5
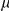
l/min. The 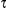 is maximum in the center of the channel, and equals 0.35 Pa for 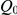, and 0.001 Pa for 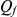.

The delivery of 21.4 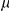
M trypsin (0.05%) through the first of the three lanes (Fig. 2A) denudes the sheet without killing cells (Fig. 2B). During delivery, the diffusion of trypsin (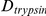 = 2.1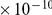 m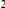/s) into the adjacent lane is limited (Peclet Number, Eq 1, 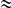 768), as the concentration gradient is sharp (Fig. 2C). This is quantified by the mixing zone, 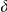, calculated at approximately 36 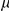
m (Fig. 2G) (in the vicinity where the subsequent migration following trypsin treatment is measured, roughly 1 mm from the point where the two fluids first meet). In addition, the gradient in functional trypsin is sharper than its concentration profile indicates, as cell media contains factors which inactivate it. As a cell diameter is on the order of 20 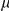
m we can be reasonably certain that cell-cell and cell-matrix contacts are not impaired beyond a single cell diameter in the remaining sheet. This is confirmed by immuno-fluorescence of the cell-cell junctional protein E-cadherin, which is intact in cells immediately perpendicular to the first row of cells (Fig S1).

After trypsin denudes the sheet, 10 mM hydrogen peroxide (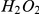) is perfused through the same inlet (Fig. 2D). It then diffuses into the adjacent lane that contains cell media, and induces apoptosis in the leading edge cells that reside there (Fig. 2E) (similar to the latent apoptosis during a physical scratch, Fig S2). The diffusion constant for 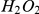 (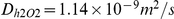) is higher than for trypsin, which generates a broader concentration profile (Fig. 2F). Thus, the mixing zone is wider than for trypsin, bathing submarginal cells in a low concentration of 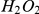 (Fig. 2G). The initial flow, 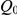 of 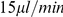, induces a mechanical shear stress along the cells of 0.34 Pa (Fig. 2H). When the flow is reduced to 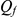 of 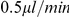, the stress drops to 0.001 Pa (Eqns 2,3,4,5).

### Denudation Interrupts the Balance in Tension Across the Epithelial Sheet

**Figure 3:**
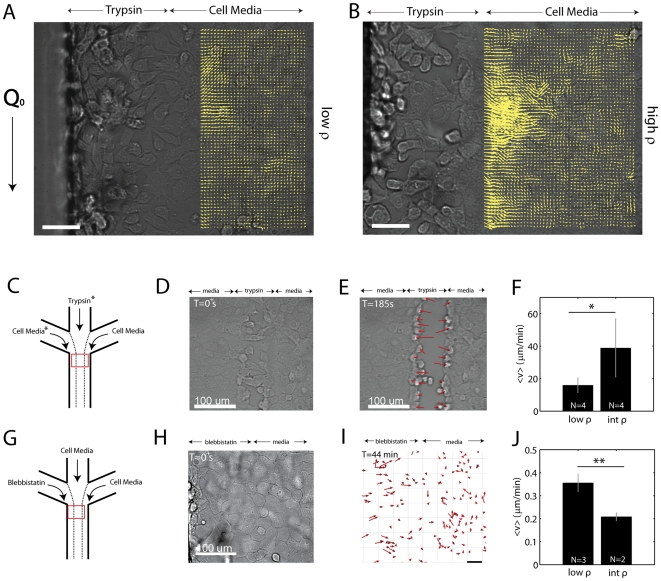
Denudation Disrupts the Balance of Tension in the Sheet. (A,B) Displacement field of the retraction of an epithelial sheet during the enzymatic cleavage of cells from the left lane. Retraction is shown by the direction of the yellow vectors that are plotted over top of the submarginal region of the sheet. A low density sheet (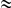 2000 cells/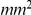) is shown in (A) and a high density sheet (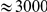 cells/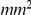) is shown in (B). In both cases, retraction occurs for multiple cell diameters. The retraction occurs perpendicular to the flow, 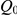. Scale bar is 50 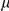
m. Images are in brightfield. The spacing between grid points is 8.6 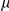
m. (C) Schematic of the experimental setup in (D-F). Trypsin is delivered through the central channel or the left channel. We group the results in both cases (*). (D) During trypsin treatment, cell bonds break and cells elongate in the direction of movement. (E) After a few minutes, the sheet is cleaved, and there is a retraction of the border cells perpendicular to the flow. Images are in brightfield. (F) The retraction of the border cells is shown to be dependent upon density, where cells are grouped broadly into two regimes, low density (below 2000 cells/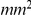) and intermediate density (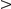
2000 cells/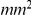). There is a clear density dependence on the retraction (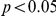). (G) Schematic for the experimental setup in (H–J). 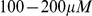 blebbistatin is delivered in the leftmost channel to disrupt actomyosin contractility (with cell media in the other channels). (H) Brightfield image of a confluent epithelial sheet during blebbistatin treatment. (I) Retraction at the blebbistatin/media interface at T = 44 min (Movie S3). Red Arrows show cell displacement. Scale bar is 50 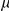
m. (F) Density dependence of retraction with blebbistatin treatment (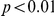). Images are in brightfield.

Within minutes of the introduction of trypsin, cell-cell and cell-matrix bonds are broken, and there is a retraction of the remaining sheet perpendicular to the new ‘ wound’ (Fig. 3A,B). This retraction consists of small displacements of fiducial markers in brightfield images of the epithelial sheet, in the direction opposite of the wound. These displacements are extracted using Particle Image Velocimetry (PIV). The displacement field shows net displacements within the sheet over a hundred microns from the leading edge. This range is much larger than the mixing zone, 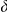, for trypsin at the trypsin/media interface, and thus is not a result of compromised cell-cell contact in the submarginal region of the sheet. Furthermore, the magnitude of these displacements are related to the cell density (Movie S2). Epithelial sheets of approximately 2000 cells/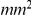 (Fig. 3A) show smaller vector magnitudes than do sheets at approximately 3000 cells/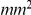 (Fig. 3B).

We quantitatively compared the rate of retraction across density, this time using the magnitudes of the retractions at the border alone via cell tracking as opposed to PIV due to the high heterogeneity of the displacements further towards the rear of the sheet (Fig S3). We also group the retraction that occurs when trypsin is delivered from either the central lane, or from a side lane (Fig. 3C). Again, upon initial introduction of trypsin, cell-cell contacts begin to break, and cells elongate perpendicular to the direction of the flow (Fig. 3D). After this initial change in cell morphology, the cells within the first couple of rows on either side of the cleared space retract at a mean speed, 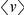 (Fig. 3E), as measured by cell tracking. Below 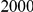 cells/mm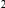, 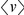  =  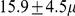
m/min. At cell densities above 2000 cells/mm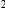, 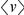 =  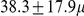
m/min (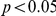) (Fig. 3F).

We also sought to test the mechanical integrity of the sheet without a denudation that disrupts cell-cell and cell-matrix bonds. To do this, we selectively inactivated cell contractility with blebbistatin, an inhibitor of non-muscle myosin II, and again measured retraction of the sheet (Fig. 3G). By delivering blebbistatin in place of trypsin (Fig. 3H), we create a gradient in cell contractility across the cell sheet, where one side of the sheet has functional actomyosin machinery, and the other side of the sheet does not.

The localized delivery of blebbistatin also results in retraction of the cell sheet perpendicular to the direction of flow (Fig. 3I, Movie S3). This retraction occurs primarily at the boundary between the two fluid streams that contain blebbistatin and cell culture medium (Fig S4). Thus, cells without functional actomyosin machinery are presumably ‘ pulled’ by the cells that do have functional actomyosin. Below 2000 cells/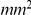, these non-contractile cells elongate perpendicular to the flow, and move at 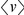 =  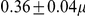
m/min. At densities above 2000 cells/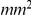, the retraction is reduced to 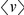 = 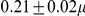
m/min, presumably due to pulling against a higher load (Fig. 3J).

### Free Space is Sufficient for Generating Collective Motion at the Border

**Figure 4:**
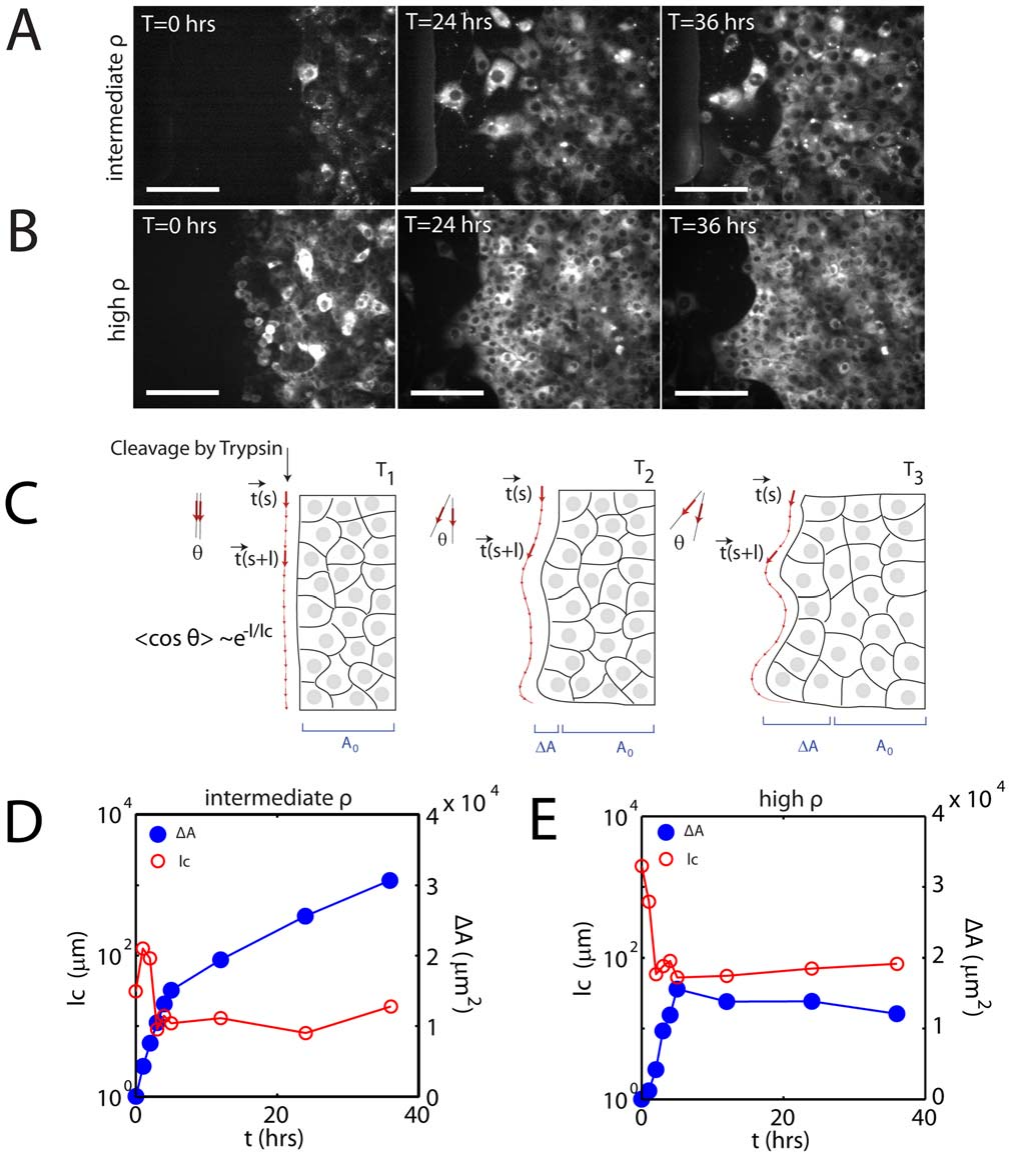
Healing is a Composite of Spreading and Migration. Timelapse of healing of enzymatically denuded sheets for (A) intermediate density (2000-3000 cells/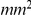) and (B) high density (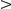
3000 cells/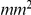). Scale bar is 100
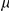
m. Cells are stained with DiI. (C) Diagram of migration that increases the overall sheet area (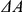), which also increases the curvature of the leading edge border. The cell sheet immediately after cleavage by trypsin (left) has a linear profile (red arrows). Thus the angle between successive vectors is small. Over time, the cell edge becomes non-uniform (middle, right). This increases the angle between successive vectors. The mean cosine decays with a characteristic length, 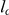. In comparing migration rates, we discount the initial acquired area, as it likely corresponds to the spreading regime, as 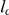 varies during this time. (D,E) Increase in acquired area, 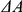 (blue, closed circle) and characteristic length, 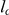 (red, open circle) for the (D) intermediate density and (E) high density cell sheet.

The healing of the epithelial sheet, whether denuded enzymatically or abrasively, begins with spreading of the leading edge cells in the direction of the wound. They subsequently migrate into the free space, followed by the submarginal cells. The spreading and motion of the cells will yield an increase in the area (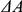) of the sheet over time. The dynamics of this movement is highly cell density dependent, and manifest in two ways. The first manifestation is in the shape of the border, the profile of leading edge cells as they spread into free space. The second, is the speed of the recovery, or the rate of migration that follows the initial spreading. Intermediate densities (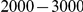 cells/mm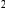) fail to develop a fully continuous border, yet migrate quickly (Fig. 4A). Highly dense sheets however (above 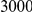 cells/mm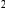), develop a very continuous border, but are slow to migrate and recover space (Fig. 4B).

The shape of the border during spreading can be measured by the characteristic length (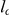), Eq 6. This is the length over which the cell border persists as straight in a particular direction. Immediately after trypsin treatment, the border is straight, and yields a high 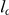 (Fig. 4C, left). Over time, the non-uniform spreading and migration of the leading edge cells adds curvature to the border (Fig. 4C, middle). This enhanced curvature will lower 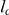. Eventually, 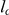 will cease fluctuating, and remain relatively constant (Fig. 4C, right). After 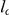 equilibrates, the area continues to rise, but at a reduced rate (Fig. 4D,E). Therefore, at this point, we approximate that there is no net spreading, and any further increase in area is primarily due to cell migration. The rate of this increase will depend strongly on density. At intermediate densities, at the onset of spreading, there is a quick drop in 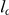 and rise in area (Fig. 4D). At densities over 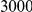 cells/mm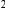 however, once initial spreading is complete, there is no further significant increase in area (Fig. 4E). We therefore choose this latter regime where 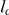 has equilibrated to distinguish the increase in area due to spreading from the increase in area due to the net migration of the tissue.

**Figure 5:**
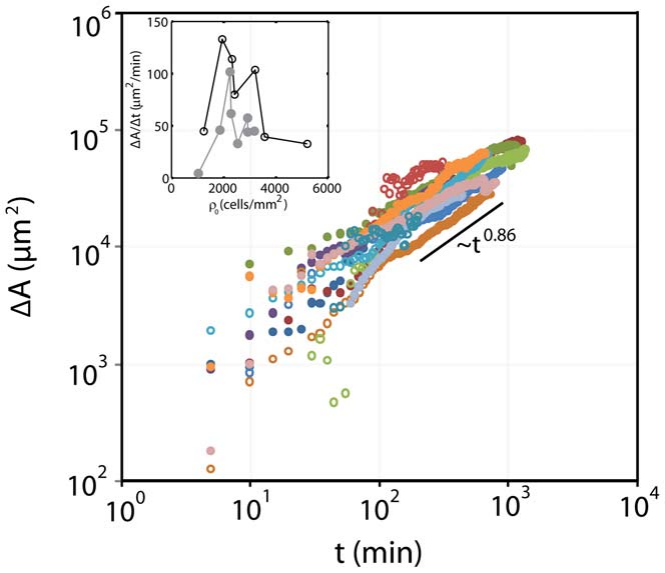
Leading Edge Dynamics are Equivalent between Abrasively Denuded and Enzymatically Denuded Sheets. Increase in area (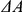) during the healing of the cell sheet (N = 15). The rate of healing, 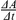 for cell sheets of different densities (inset). Solid symbols: microfluidic assay. Open symbols: scratch assay.

We contrast the dynamics of this second regime to the classical scratch assay. In both assays, the healing of the epithelial sheet increases as a power law at long times, 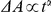, for 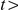 3 hrs (past the equilibration of 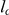 as determined from the samples in Fig. 4). They are both sub-linear with an exponent, 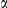 of approximately 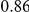 (Fig. 5). Thus, the scaling behavior is equivalent between modes of denudation (scratch: 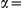 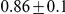, trypsin: 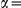 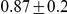). Quantifying the rates however, shows a distinct difference (Fig. 5, inset). Here, the magnitudes of the initial rates, 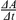, are both bimodal with density. Cells at sub-confluent densities scatter, with no significant increase in overall area. At densities above 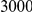 cells/mm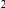, again, movement is restricted. This leaves an intermediate regime where the speed of migration is at a maximum. Comparing both assays in this regime demonstrates an elevated rate for the scratch assay. On average, at intermediate densities (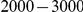 cells/mm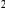), the rate of migration is nearly twice that of the enzymatic denudation (scratch: 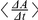 = 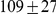 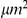/min, trypsin: 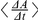 = 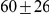 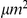/min, 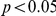).

While the progression of the leading edge, as measured by the increase in acquired area over time scales similarly between the enzymatically denuded, and the abrasively denuded sheets (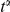), there is a clear rate difference between the two (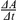).

### Rear Motility is Reduced in Enzymatically Denuded Epithelial Sheets

**Figure 6:**
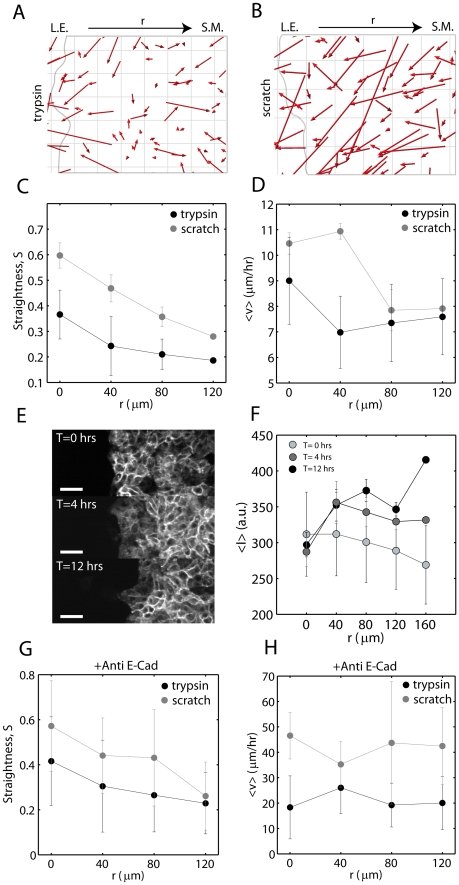
Velocity and Persistence are Hindered in the Submarginal Region of Enzymatically Denuded Sheets. Displacement Vectors (by cell tracking) for sheets after enzymatic (A) or scratched (B) denudations (red arrows). L.E.: leading edge, S.M.: submarginal cells. The leading edge is hand drawn in gray. Grid size is 20 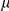
m. Straightness (C) and Speed (D) of cell movement as a function of distance from the (initial) leading edge, 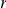 (N = 6). (E) Immunostaining of E-cadherin at T = 0, 4, and 12 hours post cleavage (N = 3). Scale bar is 50
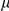
m. (F) Fluorescence intensity as a function of 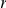. Straightness (G) and Speed (H) as a function of 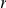 for sheets blocked with 200
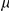
g of anti-E-cadherin antibody (N = 2).

To pursue an explanation for the reduced speed of leading edge progression in the enzymatically cleaved cell sheets, we performed a more thorough investigation into the pattern of motility of the submarginal cells. As all cells share cell-cell contact, the reduced motility of the rear may oppose outward movement of the leading edge. Therefore, by tracking the motion of the submarginal epithelial cells (Fig. 6A,B), and segregating their trajectories as a function of the distance from the leading edge, we can test this hypothesis.

We measure the speed 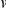, and straightness 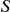 (Eqn 7) of the trajectories of individual cells in epithelial sheets of intermediate density (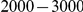 cells/mm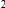, where 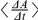 was at a maximum), up to 24 hours after denudation. We then take the average of these two statistics for cells at a distance 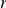 (in 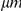), from the leading edge (at t = 0 hrs). Within half of a 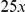 field of view, we can analyze up to 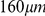, or roughly 10 rows of cells. For statistical analysis, we group cells together in intervals of 40
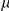
m from the leading edge. Thus an 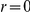 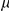
m constitutes the leading edge cells (cells within the first 40
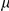
m), followed by all cells 40
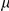
m behind the leading edge (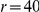 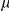
m) and so on.

For all 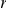, the motion of cells in the model wound (Movie S4) is more random (small 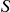) than motion in the scratch assay (Fig. 6C, Movie S5). Following denudation, cells in the model wound are less able to move in the direction of the free space. The straightness of their paths decreases monotonically with distance from the leading edge. The scratch assay also decays monotonically, but is nearly two-fold greater in magnitude than the microfluidic assay.

Cell speed immediately behind the leading edge (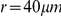) is reduced in comparison to the scratch assay (Fig. 6D). The scratch assay has no reduction in cell speed in this regime, while migration in the model wound is significantly reduced. At greater distances from the leading edge, the difference in cell speed between the two assays is lessened.

Immunofluorescence images taken at sequential time points through healing indicate increasing levels of E-cadherin in submarginal cells with elapsed time (Fig. 6E). Immediately after enzymatic cleavage, the quantity of E-cadherin is roughly equivalent at all distances from the leading edge. However, after 4 hours, there is similar localization at the leading edge, but there is enhanced localization in the rear, particularly in the vicinity of 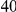 to 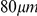 from the leading edge (Fig. 6F), consistent with the reduced motility at this margin. This pattern of localization remains constant past 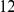 hours. Thus, given the correspondence between reduced motility and enhanced E-cadherin levels, we chose to block the ability for E-cadherin to bind between cells by using an antibody against it. The expression of E-cadherin has been shown to be inversely related to the invasiveness of epithelial cells [35]. When we introduce antibody to block E-cadherin dimerization, we see similar effects, that submarginal motility is reestablished (Fig. 6G,H). Both the straightness and speed are elevated in contrast to their unblocked state, and there is markedly increased speed overall, at all depths (N = 2), (Movies S6,S7).

**Figure 7:**
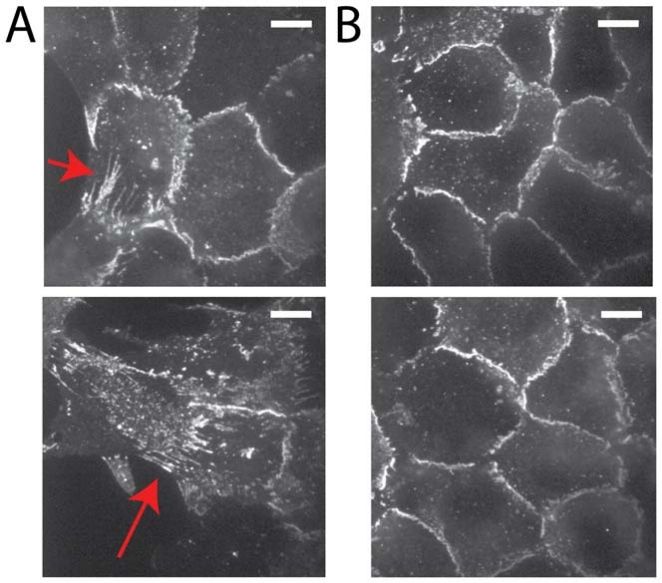
Tension at E-cadherin junctions between the leading edge and submarginal cells. Immunostaining of E-cadherin of epithelial cells 20 hrs after denudation by trypsin. (A) Two examples of E-cadherin localization at the leading edge cells that shows elongated cell-cell interface (red arrows), both in the direction of movement as well as perpendicular to the direction of movement. (B) E-cadherin in submarginal cells that does not have elongated interfaces. Scale bar is 10 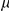
m.

The reduction in cell speed immediately behind the leading edge (40-80
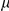
m) is consistent with the differential localization of E-cadherin between the leading and submarginal regions (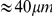). At the leading edge, E-cadherin is elongated between cells, both in the direction of the edge (Fig. 7A, top), as well as perpendicular from the edge (Fig. 7A, bottom). Distal to the edge, there is little elongation of the localization of E-cadherin (Fig. 7B).

It may be possible that the difference in the dynamics of cell migration between the two assays to the presence of a pro-migration signal that is present in cell lysate. Therefore, to further examine this difference, we utilize the same experimental setup using multiple laminar flows, to reintroduce the factors associated with cell death, still without any physical abrasion.

### ROS without Wound inhibits Collective Motion

The first approximation to an ‘ abrasion-less’ wound is the combination of trypsin to denude the sheet, and subsequently, perfuse cell lysate to simulate the cell damage that results from wounding. The lysate is created through sonication, and is expected to contain the milieu of chemical factors that result from an abrasive death. A second approximation is to engage signaling thought to influence migration more directly. The composition of lysate is ambiguous, as would be the quantity necessary to ellicit a response. As previously stated, reactive oxygen species (ROS) are thought to be involved in the promotion of wound healing. *In vitro*, they have been found transiently (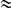 10 min) within the first 3–4 rows from the leading edge [13]. 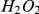 is selected for recreating this precise spatial and temporal condition, and was therefore introduced in a graded fashion across an enzymatically denuded sheet. In this sense, we simulate the leading edge exposure to ROS, still without any abrasion to the sheet. The gradient begins at a high concentration, creating an apoptotic border, and then the concentration decreases further towards the submarginal region of the sheet. Thus, the influence of peroxide may be two-fold, to induce apoptosis at high concentrations (near the leading edge), and potentially promote motion at lower concentrations (in the submarginal region). As the precise concentration of ROS to promote migration is unknown, we use this gradient such that cells are exposed to high or low concentrations, depending upon their distance from the leading edge.

**Figure 8:**
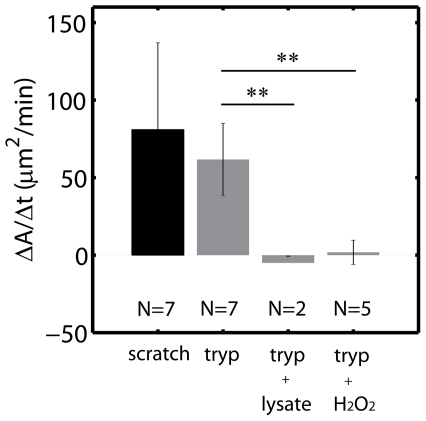
ROS Inhibits Movement in Enzymatically Denuded Sheets. Mean healing rates (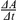) for cells immersed in lysate or exposed to a gradient in 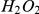 (all densities). ** indicates a p-value 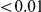. Anova Analysis in Fig S7.

Within the conditions described in this article, motion was not recapitulated with lysate or peroxide treatment (Fig. 8A,B, Fig S7). The addition of lysate or peroxide significantly inhibited outward motion of the free edge. Thus the composition of lysate and ROS was either inhibitory or damaging to the cells.

## Discussion

We present a model wound healing assay designed to separate the influence of free space from cell damage on the migration of an epithelial sheet. In this assay, we can denude a sheet enzymatically, without inducing cell death or damage. With an undamaged sheet, we show that one can then systematically study the migration in response to select chemical factors involved in the wound response.

After denudation by trypsin, the cell sheet heals in two phases. The first phase is through the cell spreading that occurs at the edge. Cell spreading is fast, and can account for the healing of small wounds (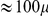
m), despite minimal or even retrograde migration of the submarginal cells (Fig S5, S6). The second phase is slower and is due to the net migration of the cell sheet. All cells, both front and rear, migrate in the direction of the wound and their velocity and the straightness of their paths decreases monotonically the further they are from the cell edge.

Two phases have been similarly observed in the migration of cell sheets after the removal of stencils that confined them [14]. With stencils, the early, fast phase of healing is described by an initial formation of columns of cells called ‘ fingers’ that quickly protrude from the edge [36]. This phenomenon accounts for a rapid healing rate (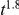) which persists for up to 20 hrs. Eventually, the cells will reach a constant velocity [6]. We draw similarities to this latter phase. Our early phase consists of the spreading of cells (1–3 hours) and healing proceeds by migration which is slow and sub-linear (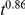). Thus within the conditions described in this article, we do not observe an extended acceleration phase. The shape of the edge is non-uniform and fluctuates, but both the geometry and dynamics are inconsistent with what is associated with fingering [12]. Thus, the formation of fingers is not a prevalent feature in our assay, nor is it clear that it is a general characteristic of the migration of mammary epithelial cell sheets [37], [38].

These dynamics that we use to characterize wound closure in our system are at an intermediate density of 2000–3000 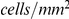. At cell densities greater than 3000 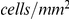, wound closure is hindered. Simplistically, one may argue that a higher density of cells yields a higher density of E-cadherin based cell-cell contact. The expression of E-cadherin has been shown to be inversely related to cell motility in cell sheets [3], [39]. Furthermore, tension is generated between cells at E-cadherin mediated cell-cell junctions [33], [34]. By extension, high levels of E-cadherin based cell-cell contact may act to impair healing in our model system through the regulation of tension. Our results show that prior to denudation and migration, when myosin is selectively inhibited, there is significant retraction of the epithelial sheet, indicative of strong tension across the cell sheet. During migration, we also see evidence for the manifestation of tension. The expression and localization of E-cadherin correlates spatially with the reduction in submarginal motility. Immediately behind the leading edge, E-cadherin junctions are elongated in the direction perpendicular to the wound. Further towards the rear of the sheet E-cadherin is elevated. The inverse relationship between E-cadherin levels and motility is confirmed through the increase in motility when E-cadherin is blocked extracellularly (20.9 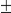 2.9 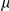
m/hr). Furthermore, if the retraction during blebbistatin treatment indeed reflects the tension in the cell sheet, and E-cadherin is involved in this tension, then the sum of the rate of retraction and the rate of migration may resemble the rate of migration with blocked E-cadherin. Indeed, these two rates are comparable (21.5 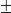 2.0 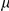
m/hr vs. 20.9 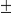 2.9 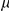
m/hr). This leads us to propose that tension may oppose the initial, outward migration of the leading edge, and is therefore in contrast to the migration that occurs in assays such as expanding colonies [22], [40], barriers [9], [13] or stencils [14] in which no sheet is denuded.

The reduction in submarginal migration in the microfluidic assay suggested the possibility that it lacked a signal that is produced by a physical scratch. Our assumption regarding this additional signal, was that it is contained in the debris that results from cell death and damage due to physical trauma. However, neither by adding cell lysate, nor by adding reactive oxygen species (ROS), could we re-establish motility of the rear cells. To the contrary, the movement in both cases was uniformly inhibited. We speculate the further reduction in velocity might be due to one or both of the following possibilities. First, the chosen lysate concentration and ROS gradient might decrease cell viability, thereby reducing migration (although not killing cells). Second, it is possible that neither the lysate nor ROS, contain the composition of chemical factors necessary for providing this pro-locomotory signal. An imbalance in the composition of chemical species may inhibit motion. Our assay provides a systematic means by which future experiments can dissect this issue.

The separation of fluids in a microfluidic chamber offers significant advancements over previous efforts to decouple the influence of free space from cell damage on the migration of epithelial sheets. Notable among these efforts is the application of physical barriers such as barriers or stencils. In contrast, our method not only eliminates cell death, but minimizes the mechanical stress that may trigger inter-cellular signaling to effect migration. The only physical interaction in our assay is through very low and transient shear stress across the cell population, for the delivery of trypsin or peroxide. Our 0.35 Pa, 10 minute shear does not impair the cells, as it is well below what is known to detach and deform [41], or alter the cytoskeletal architecture of epithelial cells [42]. However, for endothelial cells [43], [44], the response to stress is more subtle. The migration of endothelial cells is actually enhanced by as little as 0.03 Pa of continuous shear via a cone and plate [45]. Studies have further shown, that for shear stress through channels however, continuous stresses between 0.3 and 2 Pa increase endothelial wound closure [17]–[21]. Thus our initial stress is not elevated in comparison to these standards, and is not continuous, but brief. Our second stress of 0.001 Pa is continuous, but negligible. Thus, we presume the applied mechanical stress to be minimal and not bias sheet migration, as to not invalidate a comparison to the scratch assay. Further evidence of this minimal interaction is the observation that movement is reduced in comparison to the scratch assay, and not enhanced as elevated shear stress suggests.

A potential impairment of cell migration in our assay may come from the unknown effects of the proteolytic behavior of trypsin, which can result in the digestion of cell surface proteins at the leading edge [46]. However, this proteolytic behavior is restricted to the leading edge cells, whose migration is comparable to that in the scratch assay. In addition, treatment by trypsin preceded both the microfluidic assay and the scratch assay in the initial seeding of cells. As submarginal migration in the scratch assay is not hindered, we presume the recycling of cell surface proteins to be faster than the amount of time between seeding and scratching (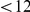 hrs on average). As our migration assays proceed for up to 36 hrs, we assume that cell surface recycling is complete, and does not significantly influence migration. Furthermore, as cell media inactivates trypsin, there should be no residual cleavage once trypsin flow is removed. Thus, impairment of the leading edge cells directly is not likely. It may be possible however, that the leading edge cells may initiate paracrine signaling during or after enzymatic cleavage, altering the migration of neighboring cells. However, convection dominates diffusion throughout the experiment, and soluble signals produced by cells are predominantly removed by flow. Diffusion of soluble signals even between neighboring cells (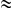 20 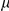
m) is extremely limited as during trypsin delivery, the Peclet Number, 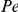 (Eq 1) (for an arbitrary globular protein of radius 10 nm) is of order 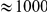, and after delivery, the flow is reduced, and 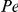 drops to 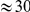.

The microfluidic aspect of our assay offers unique advantages. Through the laminar separation of fluids, any combination of chemical factors that are influential in the wound response can be controlled spatially and temporally. In this study, we were able to take advantage of this systematic method in the model reconstruction of the physical and chemical environment of a wound. We propose extending this method to explore a more extensive range of ROS, as well as other influential components of the wound response, including ATP and EGF. Furthermore, by injecting fluids of different viscosities and at different speeds, we might also be able to apply stresses selectively to different regions of the epithelium, and more rigorously investigate the influence of mechanotransduction.

These results demonstrate that the loss of spatial constraints alone induces sheet migration at the leading edge. It does not however, promote significant coordinated movement of cells in the rear of the sheet. This may be due to the differential ability for cells in the microfluidic assay to overcome significant cell-cell tension. The addition of ROS as a component of the ensemble of factors that follow cell damage in a scratch assay, failed to induce rear migration when complemented with lost spatial constraints. This implies that gradients of soluble signals of specific composition may be required to promote migration. The assay presented here can enable such a systematic reconstruction of the signaling environment after a physical wound.

## Methods

### Device Design

The microfluidic chip was fabricated by standard photolithography. The design of the channel itself was three 500 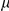
m wide channels that converge into a single 500 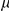
m wide channel 1 cm in length.

Three 1 mL tips served as fluid reservoirs that feed into three inlet ports. A Syringe Pump (Harvard Apparatus) was inserted at the chip outlet, pulling the fluid from the reservoirs. Flow is laminar, and leads to three separated fluid lanes across the width of the microfluidic chip (henceforth named 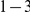 from left to right).

The diffusion between the lanes is limited, as determined by the Peclet Number, 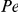 (Eq 1). This number reflects the balance of convection to diffusion. In this expression, 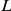 is the length-scale over which the two fluids have been in contact, 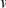 is the fluid velocity, and 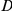 is the diffusion constant for the chemical species.
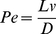
(1)


For 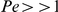, convection dominates diffusion, and there is very little mixing. For 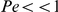, diffusion dominates convection, and the fluids mix.

We also use this expression to evaluate the extent to which soluble signals produced by cells are able to be received by their neighbors. Using 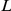 of approximately a cell diameter, or 20
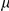
m, for 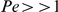, soluble signals produced by cells will be carried away by flow. For 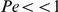, soluble signals reach cells near those that produce them.

The shear stress through the channel was calculated for rectangular channel Pouiseuille flow [47]. In this model, 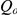 is the volumetric flow rate, what is retracted using the syringe pump. From this, we get the linear velocity, 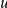 (Eq 2). The shear 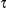 (Eq 3), is then the derivative of 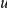, with respect to 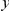, the wall axis (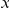 is the axis in the direction of flow, along the length of the channel, and z is the axis in the direction across the width of the channel). We are therefore interested in the shear at the bottom of the channel, 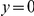, where the cell monolayer resides. The fluid viscosity is that for water, 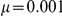 Pa s, and the pressure drop across the channel of length 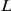 is 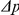 (Eq 4). The coefficients 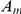 and 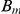 are applied to ensure the boundary conditions that velocity is zero at the walls (Eq 5). We ignore the effects of cell topology on our estimate of shear stress.
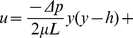
(2)

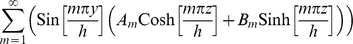


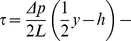
(3)

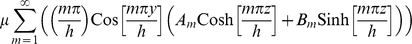


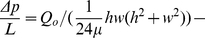
(4)

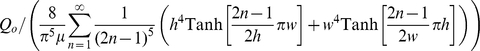


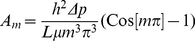
(5)

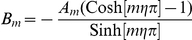



### Cell Culture

Mouse Mammary Epithelial Cells (CLS-1, ATCC, Manassas, VA) were maintained in Dulbecco's Modified Eagle's medium (Invitrogen), supplemented with 10% fetal bovine serum and 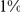 penicillin/streptomycin at 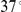
C and 5% CO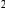 in a humidified incubator.

Fibronectin (Sigma, St. Louis) was perfused (1mg/ml and 0.2mg/ml) into the channels and incubated at 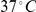 for upwards of 1 hour, and then washed out three times with media. The cells divide once every 24 hours, up to 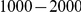 cells/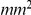 [48].

To disrupt cell-cell contact, we seeded approximately 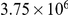 cells with 200
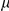
g anti-E-cadherin antibody (Invitrogen) into the chip.

### Stepwise Process of Reconstituting a Model Wound

Each experiment proceeded as follows. Step 1: reservoir 1 was filled with 0.05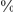 (21.4
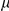
M) trypsin, and reservoirs 2 and 3 with cell culture medium. The pump was set to retract at 15
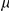
l/min until the cells have been removed, which takes less than 10 minutes. At this speed, the fluid streams containing media and trypsin are separated. This creates an interface between trypsin and media. The cleavage of cells in lane 1 is observed by brightfield microscopy (Movie S1). Subsequently, more media was added to reservoirs 2 and 3 to restrict the flow of trypsin, until the tip that originally contained trypsin could be replaced. This was done carefully, as unstable flow can result in trypsin moving into Lanes 2 and 3, and compromise cell-cell contact in the remaining cell population (Movie S10). Unstable flow may also occur if the density of cells is too high or non-uniform, as cells can occlude the inlets. Step 2: After enzymatic denudation, reservoir 1 was replaced with 10 mM hydrogen peroxide (Sigma) diluted in cell media and retracted at 15
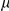
l/min to flow through lane 1, with a diffusive gradient into lane 2. This establishes the gradient in 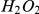 from 10 mM down to 130 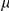
M within roughly 50
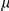
m. Thus, the sheet in lanes 2 and 3 has an apoptotic edge. The rest of the sheet was not apoptotic, and it has been suggested that this concentration is under a pro-migratory and proliferation concentration (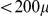
M) [49]. Step 3: Afterwards, reservoir 1 was replaced with media such that pure media fills the entire channel, and flow was reduced to 0.5
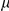
l/min. 5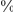 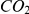 gas was fed through a tube into reservoir 2 (above the media), mineral oil added to the tops of reservoirs 1 and 3, and the entire system was maintained at 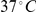 in an enclosed chamber, for the entire experiment.

For an initial fluid retraction rate of 15
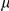
l/min, minimal for retaining a sharp gradient in trypsin or 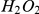, we calculated a maximum shear of 0.35 Pa, Eq 3 (Mathematica 7.0, Wolfram Research, Champaign, IL). The shear was nearly constant in the center of the channel, roughly 50
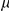
m from the walls. Thus, at this low level, the difference in the shear stress along the cells was approximately 0.1 Pa towards the walls. After less than 10 minutes of this flow, we further reduced it to 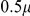
l/min for the rest of the experiment, up to 36 hrs. This corresponds to a shear stress on the order of 0.0013 Pa.

To disrupt actomyosin contractility across the epithelial sheet, 100–200
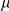
M blebbistatin (Sigma, St. Louis, MO) was delivered in place of trypsin in the above experiment for 20 minutes.

Cell lysate was produced by tip sonication for 20 minutes at 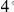
C. Lysate equivalent to approximately 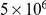 cells/mL was added to the chip. Sonication left no macroscopic debris that may clog the microfluidic channels or ports.

### Scratch Assay

The scratch assay was performed by culturing cells under identical conditions as the microfluidic assay, but in a 20mm Mattek dish (Mattek corp, Ashland, MA), and scratched with a 1mL pipette tip. The same ratio of antibody to cells used in the microfluidic assay is used for E-cadherin blocking.

### Image Acquisition and Analysis

The cells were stained with CMFDA (Molecular Probes), DiI (Molecular Probes), or by brightfield. Fluorescence images were taken at 488 nm or 568 nm, every 5 minutes for up to 1.5 days on a Zeiss Axiovert 200M/Perkin Elmer Ultraview Microscope with a 25x oil immersion lens (Plan Neofluor 25x/0.80 1 mm Korr DIC). Cells were also stained with a live/dead kit (Molecular Probes) to assay the effects of trypsin and ROS.

Imaris (Bitplane, Zurich) imaging software and Matlab particle tracking software (http://physics.georgetown.edu/matlab/) were utilized to identify the centroids of the cells stained by CMFDA and assemble time course trajectories. The tracking of brightfield images was done by blurring the image in ImageJ (NIH) to remove small detail, and tracking the regions of high intensity in Imaris. Comsol Multiphysics (Burlington, MA) was used to simulate the geometry of the microfluidic chip, and the flows induced from the syringe pump. This was done by numerically solving the Navier-Stokes Equations over the geometry of chip, and using estimates of the size of the trypsin and 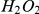 molecules to calculate their diffusivities (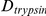 = 2.1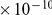 m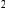/s, 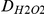 = 1.4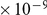 m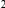/s). This allowed us to calculate their concentration profiles across the width of the channel.

Displacement fields were calculated using Particle Image Velocimetry (PIV) in MATLAB (http://www.oceanwave.jp/softwares/mpiv/). PIV was used on samples whose cell density was too high to efficiently track individual cells via fluorescence. The fields used in PIV were therefore user-defined regions in brightfield images. The field is generated through the minimum quadratic differences (MQD) algorithm. Displacement vectors were interpolated with the kriging interpolation method. The lengths of the vector fields are scaled to the size of the grid, and multiplied three-fold for visualization.

### Mathematical Methods

The shape of the free edge of the cell sheet was measured by the characteristic length, 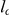, Eq 6. Similar to the ‘persistence length’ used to calculate the flexibility of polymers, we use the characteristic length analogously, to measure the ‘ flexibility’ of the leading edge as it migrates into the denuded space. Thus, we quantify how linear or non-linear the leading edge becomes over time. The characteristic length is calculated by defining a vector tangent to the free edge of the cell sheet, along the cell border. A second tangent vector, also along the cell border, separated by a distance 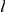 away, will have angle 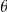 between them. For small 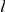, the angles are very similar. At increasing distances 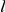, the angle 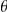 changes, and will be less correlated, as measured by the mean cosine of 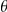. The mean cosine is an average over all separation distances 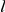, and decays as an exponential with this distance. The characteristic decay of this exponential is given by 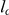.
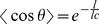
(6)


The straightness of the path a single cell takes when it migrates is measured by S, Eq 7.
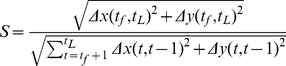
(7)


The numerator is the total distance traveled between the first time point, 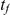 and the last time point, 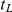. 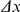 and 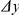 are the displacements between these two time points. The denominator is the total track length, or the sum of the displacement between each two time points taken over time, 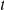. If the two distances are equal, then the path is straight, and 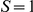. The more random the path, the closer S is to zero.

## Supporting Information

Figure S1 **Intact E-cadherin after trypsin treatment**. Immediately after trypsin is used to cleave the left lane of epithelial cells, the channel is fixed and immuno-stained for E-cadherin. There is little loss of E-cadherin even between the first and second rows of cells in low (A) or high (B) density sheets.
(TIF)

Figure S2
**Cell Death in the Classical Scratch Assay**. There is latent cell death that occurs hours after the scratch in the classical wound healing assay. This can be seen in a live/dead assay over time (left: T = 0 hrs, middle: T = 2 hrs, right: T = 5 hours after scratching). Green corresponds to live cells, and red corresponds to dead cells. Scale bar is 25 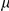
m.Initially, there are only a few red cells. There are more by the time the wound closes (B). We chose a concentration of 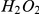 that would induce death in the leading edge cells to account for this effect. The gradient then delivers lower concentrations of 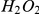 through to the submarginal cells to potentially promote movement.(TIF)

Figure S3
**Displacements in the Cell Sheet After Denudation are Heterogeneous**. Cells at varying density will demonstrate different retraction velocities when denuded by trpysin. Cell sheets at either Intermediate density (A) or High Density (B) show vector displacements opposite the wound (C,D). Images are taken in brightfield. The scale bar is 100 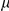
m. The vector fields are displacements taken over 30s. (E,F) Amplified region outlined by a red box from (B,C). The black line is 21 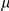
m. (D,E) Retraction velocities mapped in a contour plot. One can see that the lengthscale of retraction is significantly longer for the high density than for the low. In the main text cell tracking was used to measure cell velocity of cells at the border.(TIF)

Figure S4
**Displacements in the Cell Sheet After Blebbistatin Treatment are Heterogeneous**. Cells at varying density will demonstrate different retraction velocities when denuded by blebbistatin treatment. Cell sheets at either Low density (A) or High Density (B) show vector displacements opposite the blebbistatin at the interface between the two fluid streams (C,D). The scale bar is 100 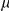
m. The vector fields are displacements taken over 3 minutes. (E,F) Amplified region outlined by a red box from (B,C). (D,E) Retraction velocities mapped in a contour plot. In the main text, cell tracking was used to measure the displacement of the particle, as cell division, and ‘rotating’ nuclei can throw off the velocities measured by PIV.(TIF)

Figure S5
**Wound Closure Can Occur Through Cell Spreading**. (A) An epithelial sheet denuded by trypsin, at over 3000 cells/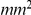 heals primarily through cell spreading at the periphery (pink: open space). (B) Taking small windows of time (4 min) and plotting the displacement fields of epithelial sheets closing the ‘model’ wound in the microfluidic assay for the sample in (A), in the region outlined in red. Much of the net migration is retrograde to the wound, yet the wound still closes due to the spreading and outward motion of the leading edge alone. We consider the healing that corresponds to Fig. 4E as this type of movement. This data set corresponds to Movies S8 and S9.(TIF)

Figure S6
**Retrograde Flow at High Cell Density Depends on Cell Density**. For epithelial sheets below roughly 2000 cells/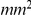, there is no significant retrograde movement (A). When cell density approaches 2500 cells/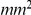, movement is in part retrograde to the wound (B). For this sample, 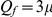
l/min, and not 0.5
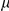
l/min like the others. For cell density above 3000 cells/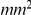, there is significant retrograde movement (B). This effect is also observed in Fig S5.(TIF)

Figure S7 **Anova Analysis of Healing Rates**. One-Way Anova for the sheets denuded by trypsin (3 independent samples: trypsin alone, lysate, and ROS) in Fig. 8. The acronyms are as follows. SS: sum of squares; df: degrees of freedom; MS: Mean-Square; F: F-test statistic; P: p-value. HSD corresponds to the absolute difference between any two sample means to have the designated significance in brackets.
(TIF)

Movie S1 **Trypsin Flow Denudes the Epithelial Sheet**. The left lane is delivering 0.05% trypsin, which enzymatically cleaves cells from the region, leaving cells treated with cell culture medium (middle and right lanes) untouched. Images are taken in brightfield.
(AVI)

Movie S2 **Trypsin-Induced Retraction is Faster at High Cell Density**. Retraction during trypsin treatment for intermediate (left) and high (right) cell density. Images are taken in brightfield.
(AVI)

Movie S3 **Selective Inactivation of Cell Contractility Results in Retraction**. The left lane is delivering blebbistatin, and the other lanes deliver cell media. After a few minutes, the cells on the left side elongate and move towards the right hand side. Images are taken in brightfield.
(AVI)

Movie S4 **Migration following Trypsin Treatment**. After the left side of the channel has been denuded by trypsin treatment, we observe migration of the epithelial sheet into the free area. The cells are stained with CMFDA.
(AVI)

Movie S5 **Migration following a Scratch**. Cells migrate to recover space after a wound has been created through scratching with a 1mL pipette tip. The cells are stained with CMFDA.
(AVI)

Movie S6 **Migration of Cells with Blocked E-cadherin following Trypsin Treatment**. Antibody against E-cadherin was added when cells were seeded into the channel. Trypsin was then delivered to the left portion of the channel to denude space. Cells largely scatter as they migrate to recover the space. The cells are stained with CMFDA.
(AVI)

Movie S7 **Migration of Cells with Blocked E-cadherin following a Scratch**. Antibody against E-cadherin was added when cells were seeded into a 20mm dish. The sheet was then scratched with a 1mL pipette tip. The cells are stained with CMFDA.
(AVI)

Movie S8 **Retrograde Flow following Trypsin Treatment of a Dense Epithelial Sheet (I)**. Time course of cells migrating to close the denuded space after the central region was evacuated with trypsin. The recovery of space is predominantly achieved through cell spreading, as there is motion of submarginal cells in the direction opposite the wound. A slower timecourse is shown in Movie S9. Images are taken in brightfield.
(AVI)

Movie S9 **Retrograde Flow following Trypsin Treatment of a Dense Epithelial Sheet (II)**. This is the same data as in Movie S8, but slowed down, and shown with greater time resolution for a brief period of time. In it, we can see the motion of cells retrograde to the wound (left side). The data in these two movies is shown in Fig S5. Images are taken in brightfield.
(AVI)

Movie S10 **Migration with Less Cell Contact**. Occasionally, the cell sheet migrates without maintaining significant cell-cell contact, as it does in this movie. We presume that this is due to unstable flow of trypsin, which then briefly compromised cell-cell contact. This can occur if the inlets had become partially occluded with cells. Alternatively this can happen if during the trypsin treatment, somewhere upstream from where we are imaging, cells are cleaved from the surface in large groups, also altering the flow profile, causing downstream digestion of cell-cell contacts. Cells are stained with CMFDA.
(AVI)
