# Supplementary figures and images for: Tension, Free Space, and Cell Damage in a Microfluidic Wound Healing Assay
Source: PLoS One. 2011 Sep 6;6(9):e24283. doi: 10.1371/journal.pone.0024283 (PMC3167843; doi:10.1371/journal.pone.0024283)

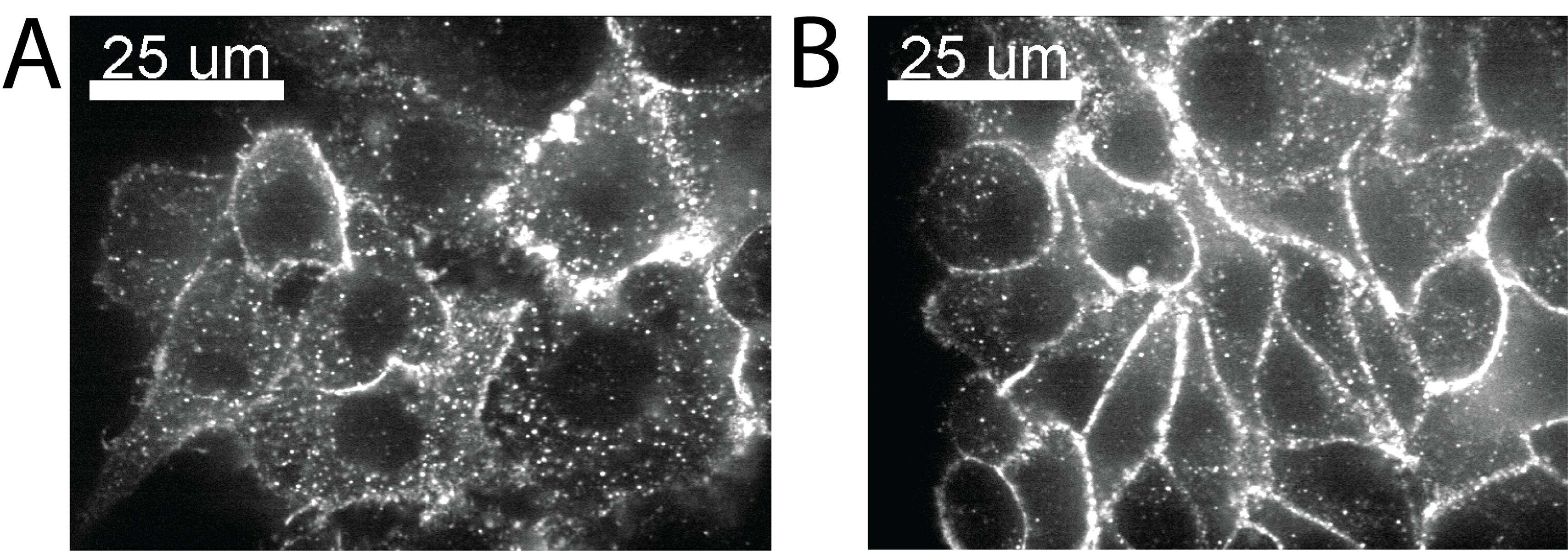

Supplement: Figure S1 — Intact E-cadherin after trypsin treatment. Immediately after trypsin is used to cleave the left lane of epithelial cells, the channel is fixed and immuno-stained for E-cadherin. There is little loss of E-cadherin even between the first and second rows of cells in low (A) or high (B) density sheets. (TIF) [file pone.0024283.s001.tif]

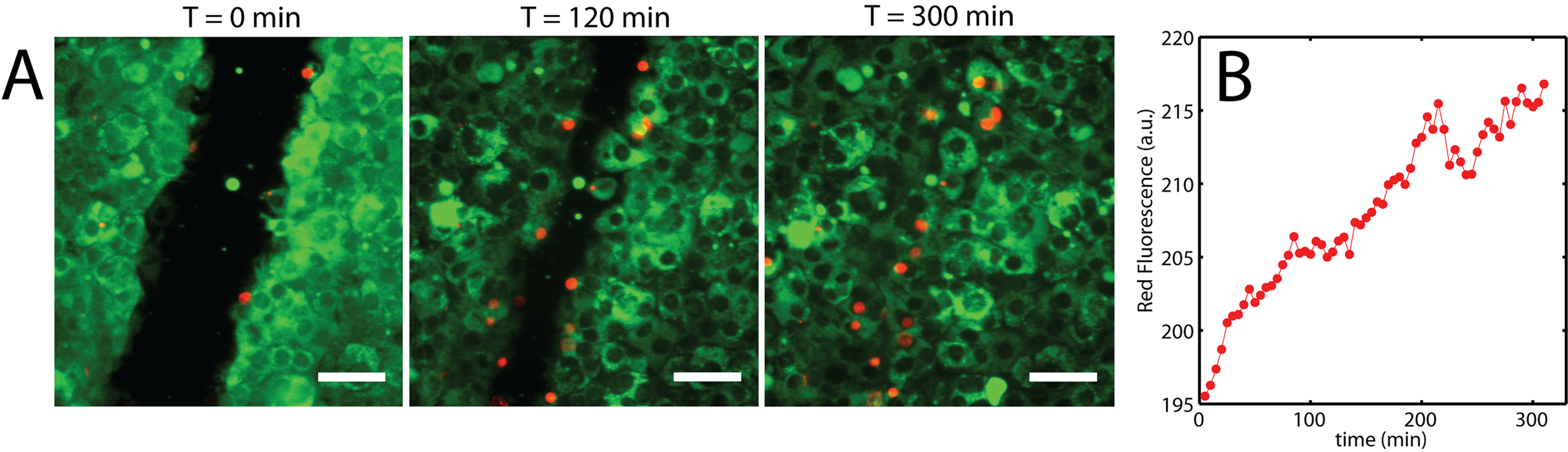

Supplement: Figure S2 — Cell Death in the Classical Scratch Assay. There is latent cell death that occurs hours after the scratch in the classical wound healing assay. This can be seen in a live/dead assay over time (left: T = 0 hrs, middle: T = 2 hrs, right: T = 5 hours after scratching). Green corresponds to live cells, and red corresponds to dead cells. Scale bar is 25 m.Initially, there are only a few red cells. There are more by the time the wound closes (B). We chose a concentration of that would induce death in the leading edge cells to account for this effect. The gradient then delivers lower concentrations of through to the submarginal cells to potentially promote movement. (TIF) [file pone.0024283.s002.tif]

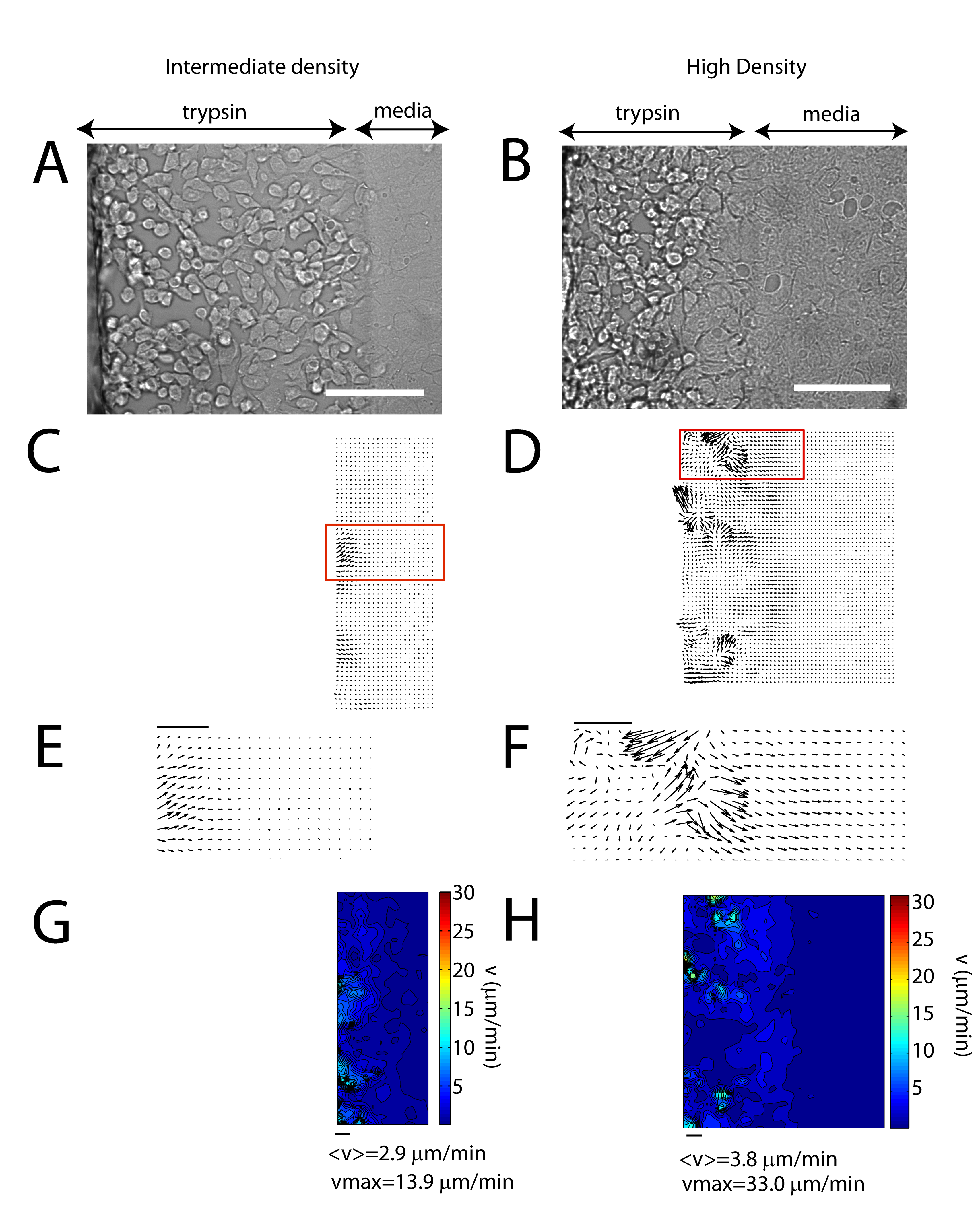

Supplement: Figure S3 — Displacements in the Cell Sheet After Denudation are Heterogeneous. Cells at varying density will demonstrate different retraction velocities when denuded by trpysin. Cell sheets at either Intermediate density (A) or High Density (B) show vector displacements opposite the wound (C,D). Images are taken in brightfield. The scale bar is 100 m. The vector fields are displacements taken over 30s. (E,F) Amplified region outlined by a red box from (B,C). The black line is 21 m. (D,E) Retraction velocities mapped in a contour plot. One can see that the lengthscale of retraction is significantly longer for the high density than for the low. In the main text cell tracking was used to measure cell velocity of cells at the border. (TIF) [file pone.0024283.s003.tif]

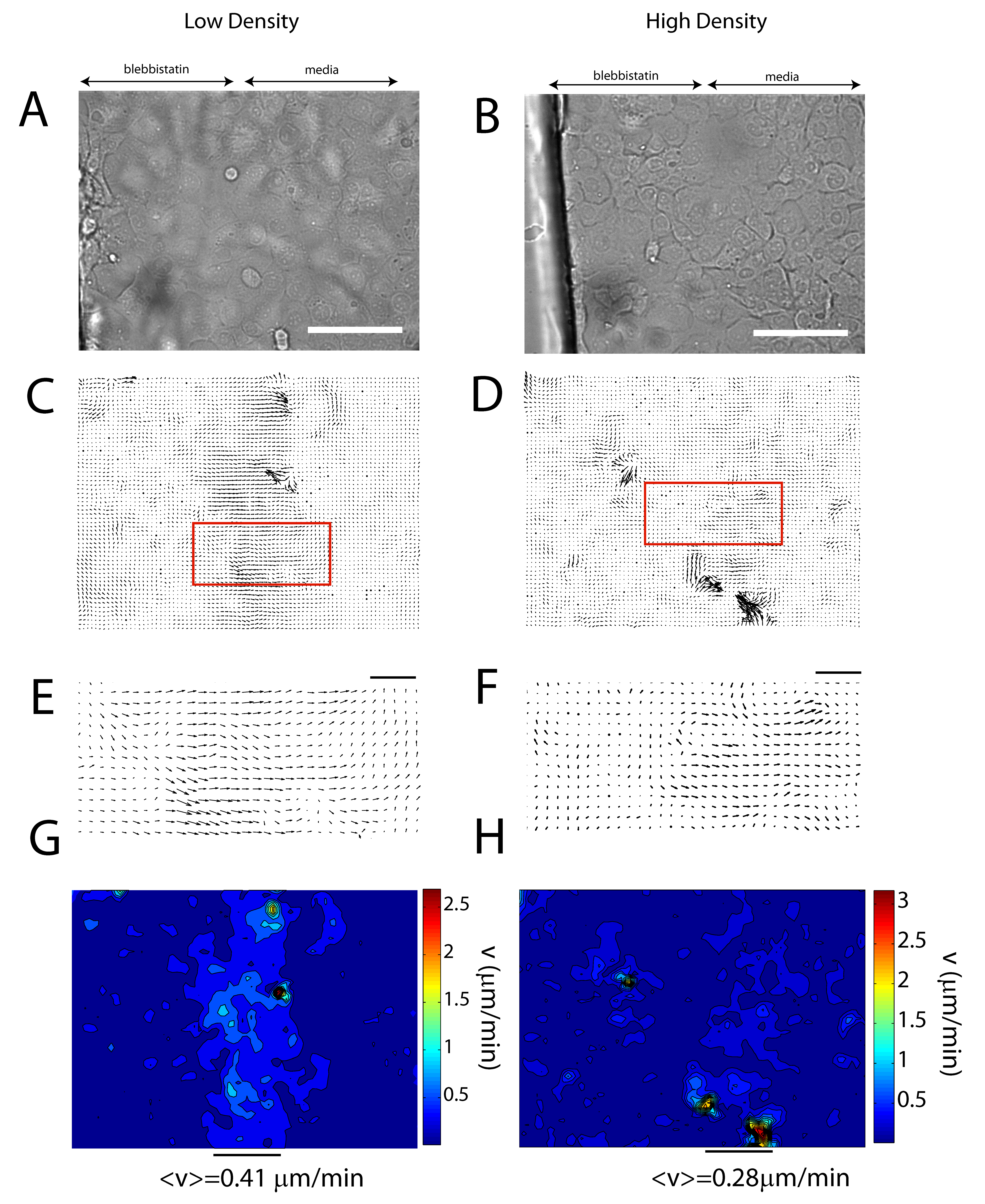

Supplement: Figure S4 — Displacements in the Cell Sheet After Blebbistatin Treatment are Heterogeneous. Cells at varying density will demonstrate different retraction velocities when denuded by blebbistatin treatment. Cell sheets at either Low density (A) or High Density (B) show vector displacements opposite the blebbistatin at the interface between the two fluid streams (C,D). The scale bar is 100 m. The vector fields are displacements taken over 3 minutes. (E,F) Amplified region outlined by a red box from (B,C). (D,E) Retraction velocities mapped in a contour plot. In the main text, cell tracking was used to measure the displacement of the particle, as cell division, and ‘rotating’ nuclei can throw off the velocities measured by PIV. (TIF) [file pone.0024283.s004.tif]

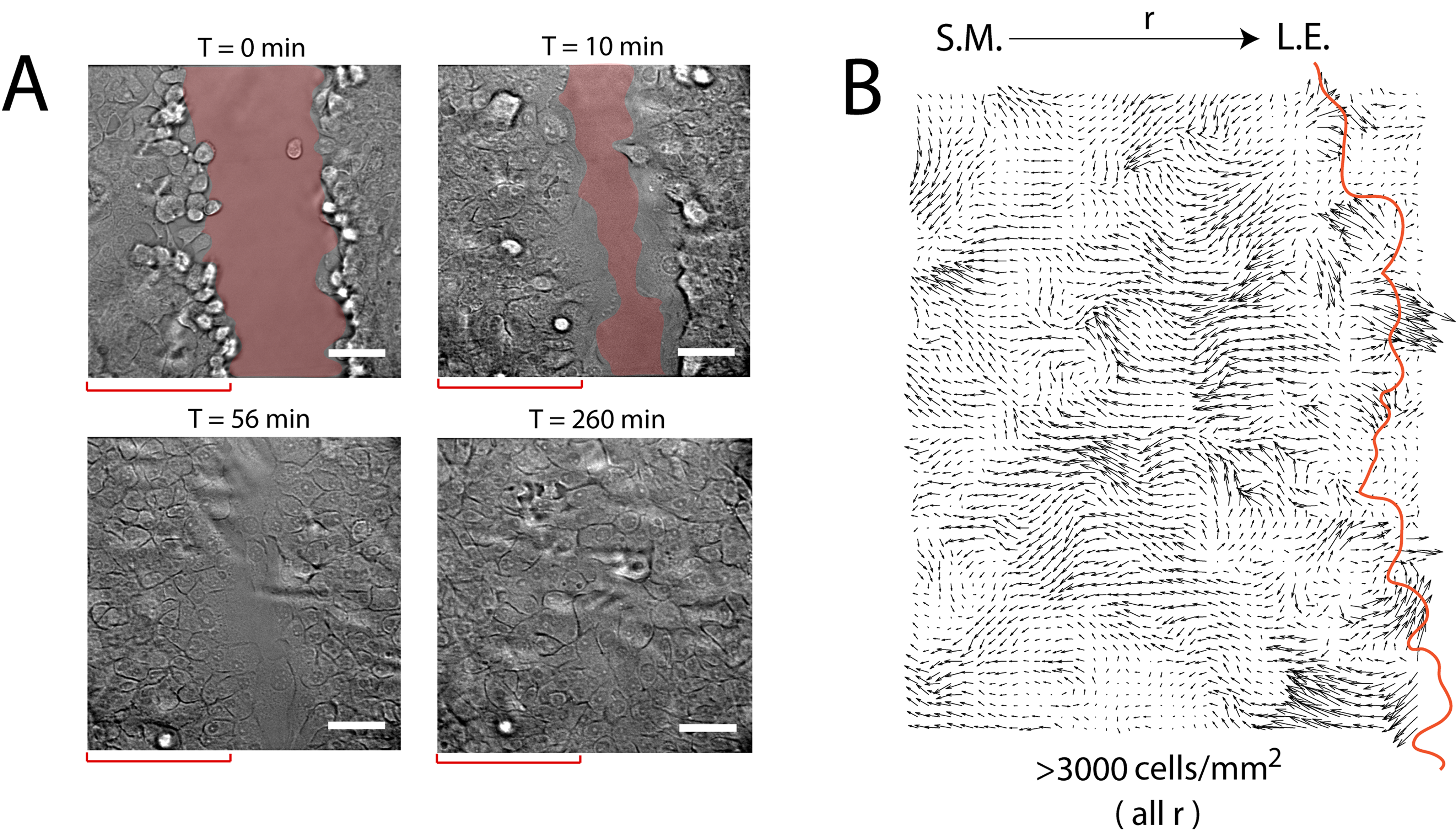

Supplement: Figure S5 — Wound Closure Can Occur Through Cell Spreading. (A) An epithelial sheet denuded by trypsin, at over 3000 cells/ heals primarily through cell spreading at the periphery (pink: open space). (B) Taking small windows of time (4 min) and plotting the displacement fields of epithelial sheets closing the ‘model’ wound in the microfluidic assay for the sample in (A), in the region outlined in red. Much of the net migration is retrograde to the wound, yet the wound still closes due to the spreading and outward motion of the leading edge alone. We consider the healing that corresponds to Fig. 4E as this type of movement. This data set corresponds to Movies S8 and S9. (TIF) [file pone.0024283.s005.tif]

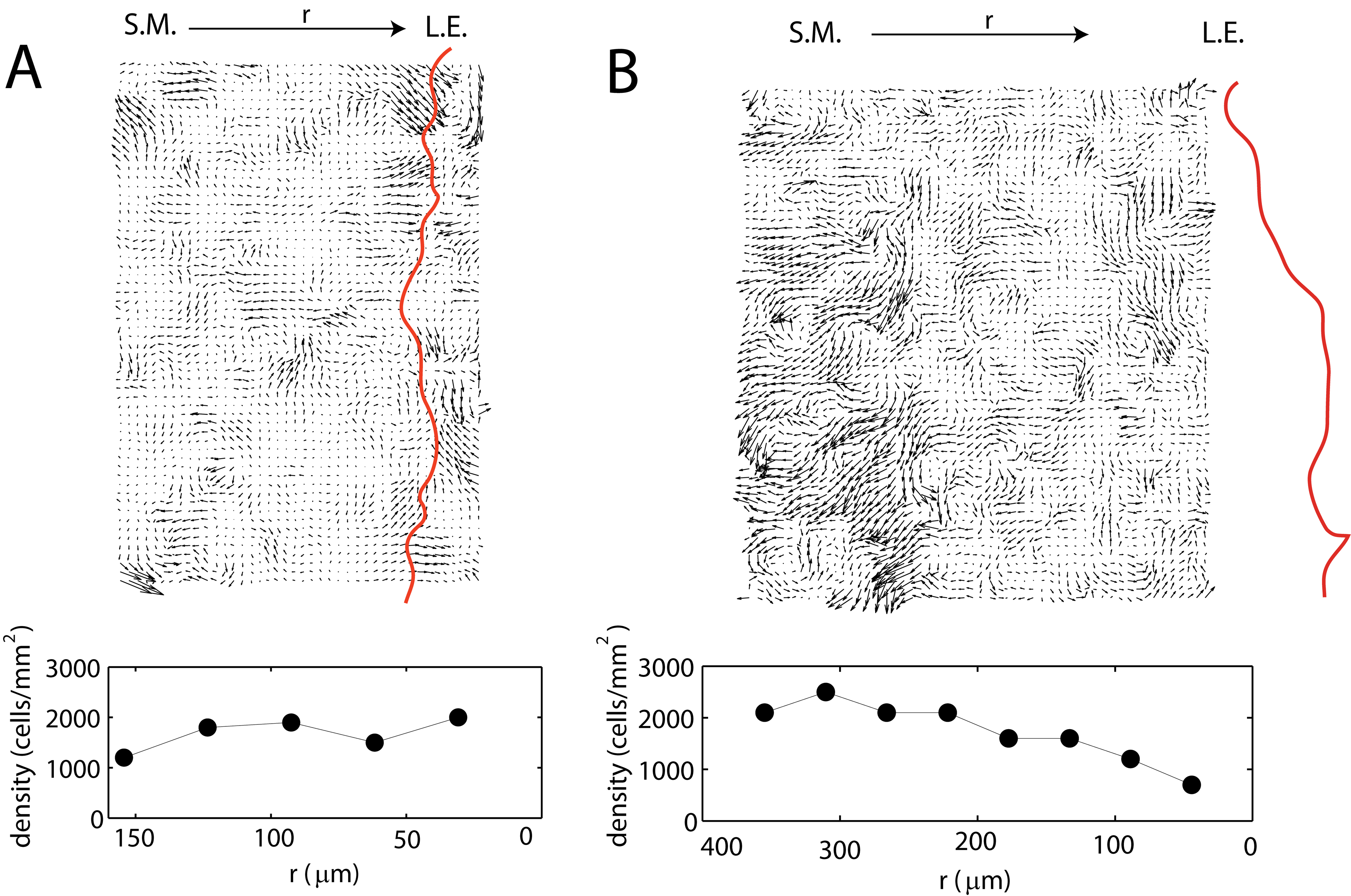

Supplement: Figure S6 — Retrograde Flow at High Cell Density Depends on Cell Density. For epithelial sheets below roughly 2000 cells/, there is no significant retrograde movement (A). When cell density approaches 2500 cells/, movement is in part retrograde to the wound (B). For this sample, l/min, and not 0.5l/min like the others. For cell density above 3000 cells/, there is significant retrograde movement (B). This effect is also observed in Fig S5. (TIF) [file pone.0024283.s006.tif]

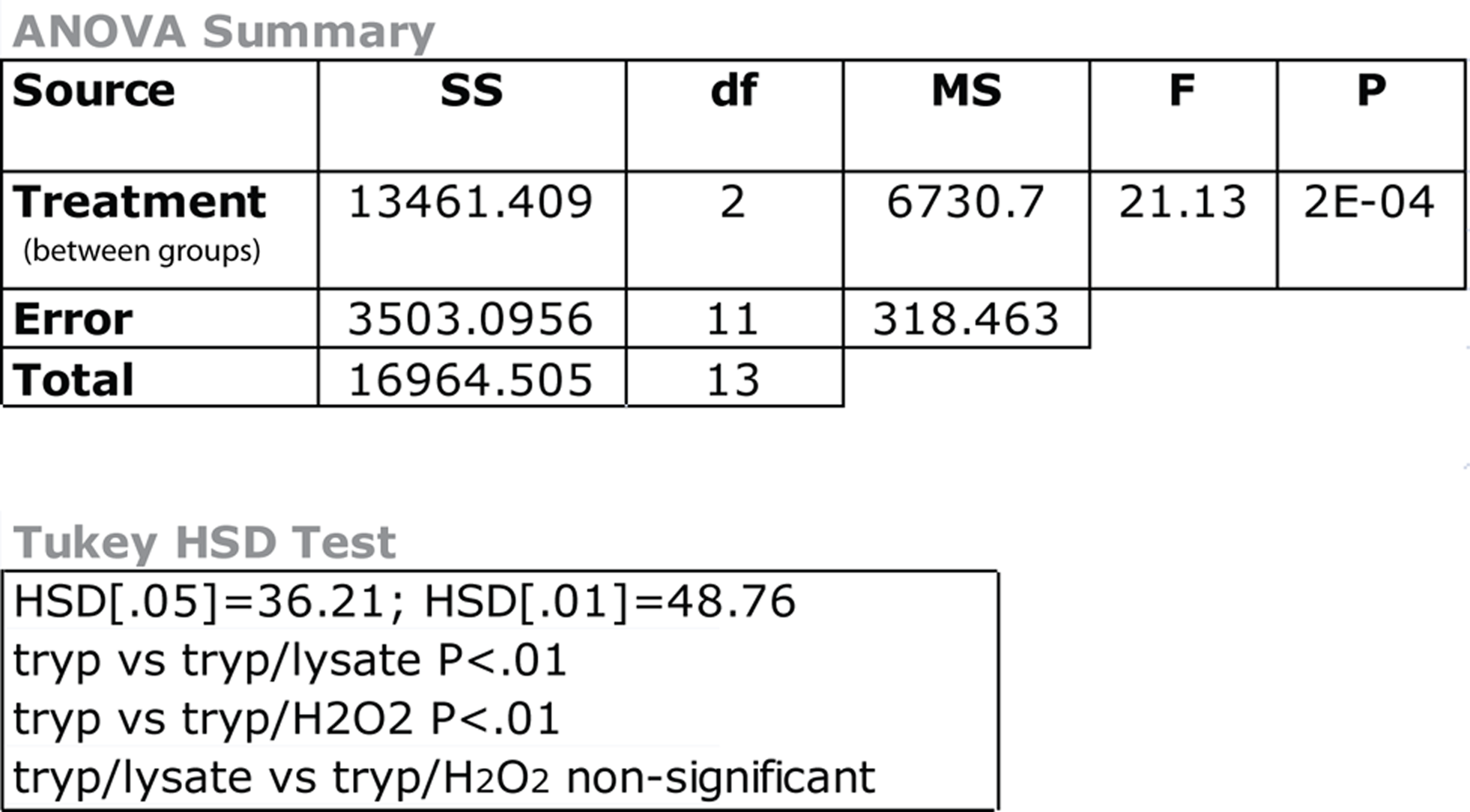

Supplement: Figure S7 — Anova Analysis of Healing Rates. One-Way Anova for the sheets denuded by trypsin (3 independent samples: trypsin alone, lysate, and ROS) in Fig. 8. The acronyms are as follows. SS: sum of squares; df: degrees of freedom; MS: Mean-Square; F: F-test statistic; P: p-value. HSD corresponds to the absolute difference between any two sample means to have the designated significance in brackets. (TIF) [file pone.0024283.s007.tif]
